# Poor Sleep Quality as a Risk Factor for Constipation Among Community-Dwelling Older Adults in Japan

**DOI:** 10.7759/cureus.46175

**Published:** 2023-09-29

**Authors:** Hiroaki Nakagawa, Taro Takeshima, Akihiro Ozaka, Sho Sasaki, Noriaki Kurita, Sugihiro Hamaguchi, Shunichi Fukuhara

**Affiliations:** 1 Department of General Internal Medicine, Fukushima Medical University, Fukushima, JPN; 2 Center for Innovative Research for Communities and Clinical Excellence (CiRC2LE), Fukushima Medical University, Fukushima, JPN; 3 Center for University-wide Education, School of Health and Social Services, Saitama Prefectural University, Saitama, JPN; 4 Department of General Medicine, Shirakawa Satellite for Teaching and Research (STAR) Fukushima Medical University, Shirakawa, JPN; 5 Section of Clinical Epidemiology, Department of Community Medicine, Graduate School of Medicine, Kyoto University, Kyoto, JPN; 6 Department of Clinical Epidemiology, Graduate School of Medicine, Fukushima Medical University, Fukushima, JPN; 7 Department of Innovative Research and Education for Clinicians and Trainees (DiRECT), Fukushima Medical University Hospital, Fukushima, JPN; 8 Department of Health Policy and Management, Johns Hopkins Bloomberg School of Public Health (JHSPH), Baltimore, USA

**Keywords:** elderly care, sleep disturbance, sleep quality, older adults, constipation

## Abstract

Background

Older adults commonly experience both sleep disturbances and constipation. Pathophysiological mechanisms such as inhibition of colonic peristalsis due to sympathetic activation associated with sleep disturbances have been postulated. Here, we aimed to assess the temporal association between the degree of sleep quality and the incidence of constipation.

Methods

We conducted a prospective cohort study of independent community-dwelling older adults aged ≥75 years (the Sukagawa Study). Using a self-administered questionnaire inquiring about awareness of own constipation or the use of laxatives in 2019 and 2020, we determined the onset of constipation. The Japanese version of the Pittsburgh Sleep Quality Index (PSQI) was used to measure sleep quality in 2019. The global PSQI score was divided into quartiles. We analyzed the association between the degree of sleep quality and the incidence of constipation using logistic regression models.

Results

Overall, 1,696 participants without constipation at baseline were analyzed after 1 year, of whom 823 (48.5%) were male. The mean age of participants was 79.9 years. In total, 191 participants (11.3%) developed constipation. The median (interquartile range; IQR) global PSQI score was 4 (2, 6). According to the quartiles of the global PSQI scores (0-2, 3-4, 5-6, and ≥7), 35 (7.8%), 55 (11.3%), 48 (12.8%), and 53 (13.8%), respectively, developed constipation. Compared to those with global PSQI scores of 0-2, the odds ratios, adjusted by age, sex, smoking status, alcohol status, educational level, working status, exercise, and medical history were 1.57, 1.78, and 2.02 for participants with global PSQI scores of 3-4, 5-6, and ≥7, respectively (*p* = 0.003 for trend).

Conclusions

We identified poor sleep quality as a new risk factor for developing constipation in independent, community-dwelling, older adults aged ≥75 years.

## Introduction

Constipation is a relevant symptom in older adults [1] that encompasses decreased bowel movements, straining, hard stools, a sensation of incomplete evacuation, and anorectal obstruction or blockage [2]. The importance of constipation is underscored by a reduced quality of life (QOL) to a degree similar to common chronic diseases such as diabetes mellitus, rheumatoid arthritis, and coronary heart disease [3], as well as by its potential for adverse acute complications such as rectal ulcer, ischemic colitis, fecal impaction, bowel obstruction, and bowel perforation.

Besides, sleep quality is an important construct from the population health point of view and comprises two aspects: objective (sleep duration, sleep latency, number of arousals) and subjective (depth, restfulness) [4]. Poor sleep quality is clinically recognized as sleep disturbance, and the prevalence of sleep disturbances accounts for 30.5% among community-dwelling older adults, according to a meta-analysis and systematic review [5]. One might hypothesize that poor sleep quality causes constipation from pathophysiological standpoints, through inhibition of gastrointestinal peristalsis and a decrease in colonic propagating sequences. [6] However, these factors, such as increased sympathetic nervous system activity [7] and disruption of circadian rhythms in the gastrointestinal tract [8,9], have insufficient support for the hypothesis from an epidemiological approach involving community-dwelling older adult populations.

Several studies have examined the association between measures related to sleep quality and constipation among populations including older adults [10-13]. In China, a study reported a correlation between sleep quality and constipation-related QOL including only constipation cases [10]; whereas, the association between sleep quality and constipation in another study included only female adults [11]. The evidence for this association is insufficient in Japanese studies because some studies showed an association between sleep disturbances and constipation whereas others did not; also they included only individuals with diseases [12,13]. More importantly, none of these studies can support the contribution of poor sleep quality to constipation because they were cross-sectional studies.

Therefore, to demonstrate a temporal association between poor sleep quality and constipation among older adults, we analyzed the association between sleep quality and the incidence of constipation over a 1-year period using data from the Sukagawa study involving independent, community-dwelling, older adults aged ≥75 years.

## Materials and methods

Study design and participants

This was a cohort study using data from the Sukagawa Study collected in 2019 and 2020. The Sukagawa Study is a prospective, community-based, cohort study conducted annually since 2015 in Sukagawa City, Fukushima Prefecture, Japan; detailed information regarding this study has been reported previously [14]. The participants were older adults aged ≥ 75 years living independently at home; we excluded those who had lost their independence based on their Japanese long-term care insurance certification status (defined as care needs level 3 or higher) as well as those who were hospitalized. We followed participants whose baseline data were measured in 2019 for 1 year.

The Internal Ethics Review Board of Fukushima Medical University School of Medicine approved this study design and its protocols, which followed the tenets of the Declaration of Helsinki (registered approval number: 2975). We obtained written informed consent from all participants. The study was reported according to the STROBE (Strengthening the Reporting of Observational Studies in Epidemiology) statement.

Measurement of sleep quality

The primary exposure was sleep quality, measured by the Japanese version of the Pittsburgh Sleep Quality Index (PSQI) [15,16], one of the most common measures of sleep quality and disturbances over the past month [4,17]. The PSQI consists of the following seven components: subjective sleep quality, sleep latency, duration, habitual sleep efficiency, sleep disturbance, use of sleeping medications, and daytime dysfunction. Each component receives a score of 0 to 3, and the global PSQI score ranges from 0 to 21. The higher the global PSQI score, the poorer the sleep quality. The Japanese version of the PSQI has shown good reliability (Cronbach's alpha 0.77) and criterion-related validity. [16] In addition, the PSQI has been shown to highly correlate with other measures of sleep quality, such as clinical diagnosis of insomnia, polysomnography, and actigraphy [18].

Definition of constipation

The main outcome was the incidence of constipation over a year, and constipation was defined by awareness of own constipation or the use of laxatives. To determine constipation, we used two questions based on actual clinical practice, as used in previous studies [12,13,19]. We asked two questions. The first question was, "How long have you been constipated?" and participants selected their response from five choices: not at all, <1 month, <3 months, <6 months, or ≥6 months. The second question was, "Have you used laxatives in the last 3 months?" and participants chose their response from five choices: never, sometimes, occasionally, weekly, or daily. Participants who chose “<1 month, <3 months, <6 months, or ≥6 months" for the first question or "sometimes, occasionally, weekly, or daily" for the second question were categorized as having constipation. These assessments were conducted in both the 2019 and 2020 surveys, and the incidence of constipation over a year was defined as a response indicating constipation in the 2020 survey among participants who did not have constipation in the 2019 survey.

Confounding variables

Confounding variables were selected based on previous studies and discussions among our researchers, including age [12], sex [11,12], smoking status [11], alcohol status [11] exercise habit [20], educational history [11], current paid work [11], medical histories of cancer [21], stroke [21], myocardial infarction [13,21], and depression [21]. Participants consuming >20 g of alcohol per day (equivalent to 500 mL of beer) more than once weekly, were categorized as current drinkers. Individuals who completed high school or higher qualifications were categorized as having a high educational level. Participants who chose yes to the question asking about engaging in a light sweating exercise for at least 30 minutes at a time, two or more days a week, for a year or more, were classified as having an exercise habit.

Statistical analysis

Analyzed participants were divided into quartiles defined by the global PSQI score. Age was analyzed as a continuous variable, and the others as categorical variables. Means and standard deviations were calculated for the continuous variable and frequencies and proportions for categorical variables. The crude and adjusted odds ratios (ORs) and their 95% confidence intervals (CIs) for the incidence of constipation were estimated using logistic regression, referring to the lowest quartile of the global PSQI score as the best sleep quality group. Adjusted model 1 was adjusted for age and sex. Adjusted model 2 was adjusted for age, sex, smoking status, alcohol status, educational level, current paid work, exercise, and medical histories of cancer, stroke, myocardial infarction, and depression. To ensure the accuracy of the results of the analysis, which adjusted for variables with a high percentage of missing data, the multiple imputation by chained equation method was used to complement the missing data for the covariates by 80 imputations [22]. Statistical significance was set at p < 0.05. Statistical analyses were performed using STATA® version 16.1 (Stata Corp LP, College Station, TX, USA).

## Results

**Figure 1:**
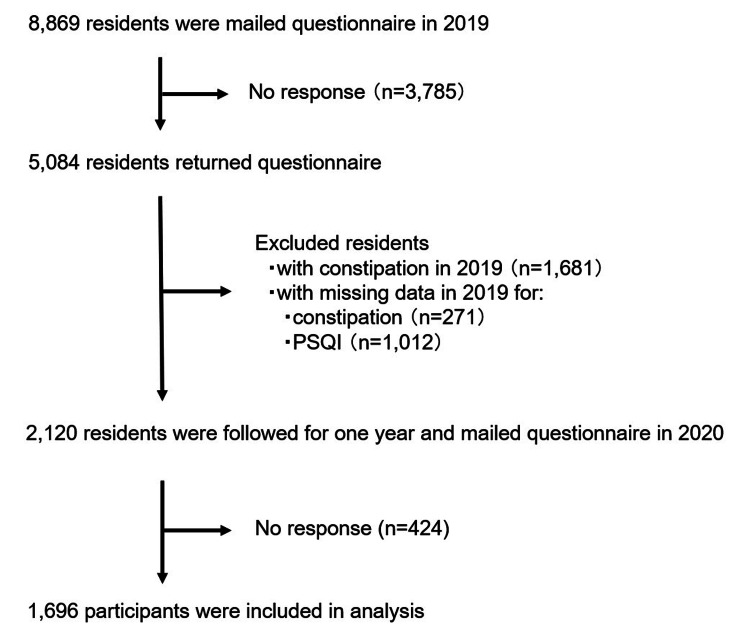
Flowchart of study participants

We sent a questionnaire in 2019 to 8,869 independent people at baseline; we received responses from 5,084 people (response rate, 57.3%). We excluded 1,681 people who had been aware of constipation or had used laxatives, 271 who had admitted missing data to the questionnaire for constipation, and 1,012 who had admitted missing data to the questionnaire for the PSQI. We followed 2,120 people for 1 year. Of these, 424 did not return the questionnaire in 2020 (follow-up rate, 80.0%); we finally analyzed 1,696 people (Figure 1).

As shown in Table 1, the mean age of the participants was 79.9 ± 4.3 years, and 823 (48.5%) were male. The median (IQR) global PSQI score was 4 (2, 6), the minimum score was 0, and the maximum score was 16.

**Table 1:** Participant Characteristics PSQI, Pittsburgh Sleep Quality Index

		Global PSQI score			
Characteristics	Total	0-2	3-4	5-6	7-
	n=1,696	n=449	n=488	n=374	n=385
Age (years) ± SD	79.9±4.3	79.9±4.4	79.9±4.3	79.9±4.3	80.0±4.3
Male, n (%)	823 (48.5)	268 (59.7)	228 (46.7)	177 (47.3)	150 (39.0)
Current smoker, n (%)	107 (6.4)	38 (8.6)	26 (5.5)	26 (7.1)	17 (4.5)
missing	34	7	15	8	4
Current drinker, n (%)	593 (35.0)	176 (39.2)	158 (32.4)	131 (35.0)	128 (33.2)
high educational level, n (%)	832 (50.7)	221 (51.4)	250 (52.7)	185 (51.0)	176 (46.9)
missing	54	19	14	11	10
Current paid work, n (%)	331 (19.6)	98 (22.1)	103 (21.2)	66 (17.7)	64 (16.7)
missing	10	5	3	1	1
Exercise habits, n (%)	231 (51.9)	71 (57.7)	59 (46.1)	41 (45.6)	60 (57.7)
missing	1251	326	360	284	281
Medical history					
Cancer, n (%)	246 (15.5)	70 (16.4)	74 (16.2)	59 (17.1)	43 (12.0)
missing	105	21	30	28	26
Cerebral infarction, n (%)	38 (2.4)	8 (1.9)	13 (2.9)	7 (2.1)	10 (2.9)
missing	142	27	42	38	35
Coronary artery disease, n (%)	139 (8.9)	31 (7.3)	35 (7.9)	36 (10.7)	37 (10.5)
missing	141	27	43	37	34
Depression, n (%)	23 (1.5)	4 (1.0)	3 (0.7)	6 (1.8)	10 (2.8)
missing	146	28	45	39	34

**Figure 2:**
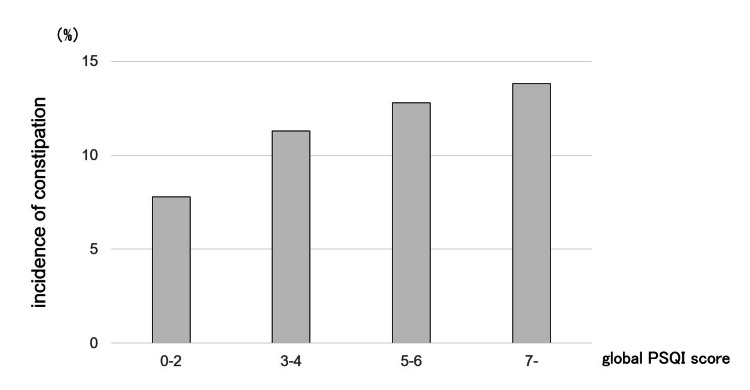
Incidence of constipation by quartile of the global PSQI score PSQI, Pittsburgh Sleep Quality Index

Of the 1,696 participants, 191 (11.3%) developed constipation during the 1-year follow-up. The number of individuals with constipation in each quartile of the global PSQI scores (0-2, 3-4, 5-6, and ≥ 7) were 35 (7.8%), 55 (11.3%), 48 (12.8%), and 53 (13.8%), respectively (Figure 2).

Association between sleep quality and the onset of constipation 1 year later

**Figure 3:**
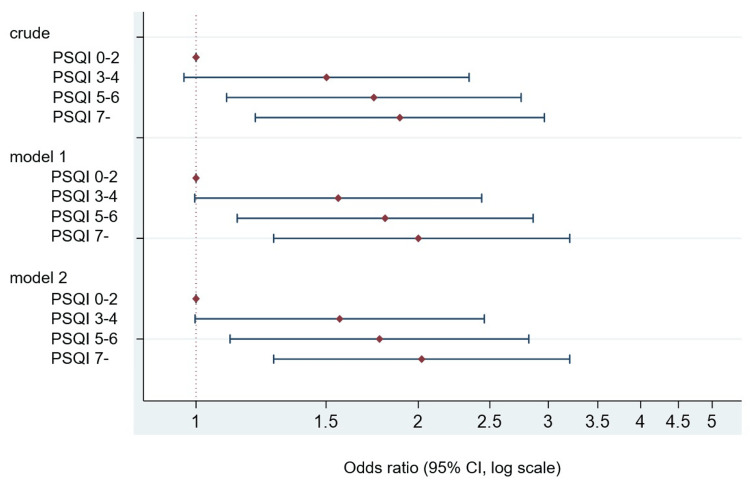
Estimated odds ratios and confidence intervals for developing constipation during the 1-year follow-up of 1,696 participants with no constipation by global PSQI score quartiles. Model 1 was adjusted for age and sex. Model 2 was adjusted for age; sex; smoking status; alcohol status; educational level; current paid work; exercise habit; and medical histories of cancer, stroke, myocardial infarction, and depression. PSQI, Pittsburgh Sleep Quality Index

The crude OR in the groups with global PSQI scores of 3-4, 5-6, and ≥7 was 1.50 (95% CI 0.96-2.34), 1.74 (95% CI 1.10-2.76), and 1.89 (95% CI 1.20-2.96; p = 0.005 for trend), respectively. The OR of adjusted model 1 (n=1,696) in the groups with global PSQI scores of 3-4, 5-6, and ≥7 was 1.56 (95% CI 0.996-2.437), 1.80 (95% CI 1.137-2.861), and 2.00 (95% CI 1.267-3.156; p = 0.003 for trend), respectively. The OR of adjusted model 2 in the groups with global PSQI scores of 3-4, 5-6, and ≥7 was 1.57 (95% CI 0.998-2.457), 1.78 (95% CI 1.11-2.821), and 2.02 (95% CI 1.271-3.199; p = 0.003 for trend), respectively (Figure 3).

## Discussion

The purpose of this study was to demonstrate the association between poor sleep quality and the incidence of constipation over a one-year period among older adults. We found that the incidence of self-reported constipation over one year was 11% among Japanese community-dwelling older adults aged ≥75 years, and that it was associated with baseline sleep quality in a dose-response manner.

The incidence of constipation among older adults presented in this study is likely the first reported from the Asian region. A previous study reporting the incidence of constipation among the older adult population has been from the United States [23]. The cumulative incidence of chronic constipation (over a median of 12 years) among residents aged ≥70 years in Olmsted County, Minnesota was 23.1% [23]. Not having a high incidence of constipation for a longer period of observation may be due to the fact that the definition of chronic constipation used in the study was deemed to be stricter than the definition of constipation in our study. That is, chronic constipation was defined by reporting at least two of four criteria, namely straining, hard or lumpy stools, incomplete evacuation, and infrequent stools - a threshold of “often” (at least 25% of the time). More reports from different geographic regions are needed to understand the incidence of constipation among older adults.

To the best of our knowledge, this study is the first to demonstrate a dose-response, temporal association between a wide degree of sleep quality and new onset of constipation limited to an elderly population, including both men and women. Several cross-sectional studies involving older adults have reported an association between sleep disturbances and constipation [10-13]. The association between sleep quality and constipation reported in a study in China only included female adults aged ≥50 years. Furthermore, sleep quality in the study was assessed simply by a single question on a 3-point rating scale [11]. Another study in China showed a correlation between sleep disturbances and constipation severity in constipated patients [10]. However, the subjects were limited to constipation cases only, and confounding factors were not addressed. The association between insomnia and constipation among Japanese inpatients may be confounded by exercise and socioeconomic characteristics [12]. A small-scale study among Japanese home medical care patients failed to demonstrate an association between insomnia and constipation [13]. Importantly, all four aforementioned studies failed to address reverse causation. In other words, they were unable to address the possibility that physical discomfort caused by constipation may result in a lack of good sleep.

Several implications from this study are potentially useful for researchers and local health policymakers. First, poor sleep quality can be screened in health checkups using an established self-report questionnaire, as used in this study. If residents with sleep disturbances can be determined; then, identification of the cause of the disturbance and the provision of treatment may help to prevent the development of constipation. Second, the dose-response relationship between sleep disturbances and the incidence of constipation (i.e., increased odds ratios along with increased PSQI global score quartile categories and significant P-values for trend) indicated that even subclinical sleep disturbances (i.e., 3 to 4 points on the PSQI global score) could contribute to the pathophysiology of constipation. The subclinical nature of this category is supported by the fact that the cutoff for primary insomnia using the Japanese version of the PSQI is 5.5 points [16]. It should be noted, however, that the lower confidence interval of the odds ratio for this subclinical sleep disorder category was slightly below 1.0 at the third decimal place level.

Several strengths of our study are worth mentioning. First, measuring a well-established measure of sleep disturbance for a large older adult population, our study was able to demonstrate its association with new onset of constipation with adjustment for key confounding factors. Second, we were able to obtain a relatively high response rate because of the cooperation of the local government. Thus, the findings of this study are considered to be representative of the community-dwelling older adults in Japan.

Several limitations are also noted in our study. First, constipation was measured at only two time points - at the beginning of the study and one year later. Consequently, misclassification may have occurred due to reporting constipation events relying on recall, as compared to prospective diary recording. Second, the interpretation of constipation was left to the individual because instruction on specific symptoms and duration was not given in the questions on constipation. Therefore, for example, an episode of no defecation for two days could be interpreted by some as constipation and others as not constipation. Third, we could not adjust for confounding factors that cause both constipation and insomnia, such as medications (e.g., antidepressants, beta-blockers, [24,25] or opioids [26]) or diseases (e.g., Parkinson's disease [27] or hypothyroidism [28]). However, given the infrequency of these diseases and adverse drug events, we do not believe failure to adjust for these factors would greatly alter the magnitude of the association between sleep quality and constipation. Fourth, due to limited resources and especially the time and expertise of the research staff [29], we measured sleep quality by a self-reported scale instead of implementing any sleep measurement devices for all the subjects. This may have led to misclassification of sleep quality. Finally, because this study was conducted among community-dwelling older adults in Japan, our findings could not be extrapolated to other countries with different lifestyles, including defecation styles.

## Conclusions

The incidence of constipation was approximately 11% during a one-year follow-up period among independent Japanese community-dwelling adults aged ≥75 years. We identified self-reported poor sleep quality as being associated with the incidence of constipation in a dose-dependent manner. Further studies are warranted to elucidate whether constipation can be prevented by improving sleep quality.
